# Unprecedented evidence for high viral abundance and lytic activity in coral reef waters of the South Pacific Ocean

**DOI:** 10.3389/fmicb.2014.00493

**Published:** 2014-09-23

**Authors:** Jérôme P. Payet, Ryan McMinds, Deron E. Burkepile, Rebecca L. Vega Thurber

**Affiliations:** ^1^Department of Microbiology, Oregon State UniversityCorvallis, OR, USA; ^2^Institute for Pacific Coral ReefsMoorea, French Polynesia; ^3^Department of Biological Sciences, Florida International UniversityMiami, FL, USA

**Keywords:** marine viruses, viral lysis, carbon cycling, coral reefs, South Pacific, microbial mortality, viral abundance, spatial and temporal variability

## Abstract

Despite nutrient-depleted conditions, coral reef waters harbor abundant and diverse microbes; as major agents of microbial mortality, viruses are likely to influence microbial processes in these ecosystems. However, little is known about marine viruses in these rapidly changing ecosystems. Here we examined spatial and short-term temporal variability in marine viral abundance (VA) and viral lytic activity across various reef habitats surrounding Moorea Island (French Polynesia) in the South Pacific. Water samples were collected along four regional cross-reef transects and during a time-series in Opunohu Bay. Results revealed high VA (range: 5.6 × 10^6^–3.6 × 10^7^ viruses ml^-1^) and lytic viral production (range: 1.5 × 10^9^–9.2 × 10^10^ viruses l^-1^ d^-1^). Flow cytometry revealed that viral assemblages were composed of three subsets that each displayed distinct spatiotemporal relationships with nutrient concentrations and autotrophic and heterotrophic microbial abundances. The results highlight dynamic shifts in viral community structure and imply that each of these three subsets is ecologically important and likely to infect distinct microbial hosts in reef waters. Based on viral-reduction approach, we estimate that lytic viruses were responsible for the removal of ca. 24–367% of bacterial standing stock d^-1^ and the release of ca. 1.0–62 μg of organic carbon l^-1^ d^-1^ in reef waters. Overall, this work demonstrates the highly dynamic distribution of viruses and their critical roles in controlling microbial mortality and nutrient cycling in coral reef water ecosystems.

## INTRODUCTION

Viruses are increasingly recognized as the most abundant and dynamic biological entities in marine ecosystems (e.g., reviewed in Fuhrman, 1999; Wommack and Colwell, 2000; Weinbauer, 2004; Suttle, 2007). Viral-mediated cell lysis can cause significant mortality of heterotrophic bacteria, cyanobacteria and eukaryotic phytoplankton (Wilhelm and Suttle, 1999; Brussaard, 2004b). Models and empirical studies have estimated that 20–50% of marine microbial communities are infected by viruses each day (e.g., reviewed in Fuhrman, 1999; Wilhelm and Suttle, 1999; Suttle, 2005, 2007). The release of organic cellular content and nutrients upon viral lysis can stimulate autotrophic and heterotrophic microbial activity (Gobler et al., 1997; Middelboe and Lyck, 2002; Weinbauer et al., 2011; Shelford et al., 2012) and increase diversity (Weinbauer and Rassoulzadegan, 2004; Motegi et al., 2013), with major effects on global biogeochemical cycles and flow of energy in the oceans (Fuhrman, 1999; Wilhelm and Suttle, 1999; Suttle, 2007). Despite their critical impact in the oceans, there is still a lack of data on the spatial and temporal dynamics of viruses and their ecological influence in marine microbial communities.

Tropical coral reefs are highly productive and diverse ecosystems yet thrive under oligotrophic conditions. Accumulating evidence also suggests that reef waters harbor abundant and active microbial communities (Moriarty et al., 1985; Gast et al., 1998; Charpy et al., 2012 and references therein) that can respond rapidly to changes in environmental conditions (Haas et al., 2011, 2013; Nelson et al., 2011; Mccliment et al., 2012). In this setting of nutrient poor conditions and high microbial abundance, viruses may play a particularly important ecological role in shaping microbial communities, with potential impacts on carbon cycling and energy transfer to higher trophic levels.

The spatial and temporal patterns of viral abundance (VA) and production have been relatively well studied in various marine environments over the past decades, but only a few studies have focused on marine viruses in tropical and subtropical reef waters (Paul et al., 1993; Seymour et al., 2005; Patten et al., 2006; Bouvy et al., 2012). These few studies have suggested that viruses are as highly dynamic and abundant as reported in higher latitude marine environments (e.g., reviewed in Vega Thurber and Correa, 2011). To our knowledge, the only study that has investigated lytic viral activity in coral reef waters estimated that lytic viruses were not a significant source of mortality for bacteria in atoll reef waters (Bouvy et al., 2012), in contrast to general findings from other marine ecosystems. More work is needed to fully elucidate the potential ecological roles of viruses in coral reef waters.

Here, we investigated viral abundance, subset composition as detected through flow cytometry (FC), and production in the south Pacific island of Moorea, French Polynesia. Particularly, we evaluated whether VA, structure, and lytic activity changed across distinct reef habitats and time. Furthermore, we used multivariate analysis to assess potential ecological factors controlling distribution patterns of VA and lytic activity; specifically, we asked whether patterns were driven by changes in trophic status of the ecosystem and/or by environmental conditions. Finally, we aimed to assess how virus-mediated mortality of heterotrophic bacteria can influence dissolved organic carbon (DOC) availability in oligotrophic reef habitats. Collectively, this novel dataset allowed us to determine whether viruses are dynamic and important players in tropical planktonic reef ecosystems.

## MATERIALS AND METHODS

### STUDY AREA

**FIGURE 1:**
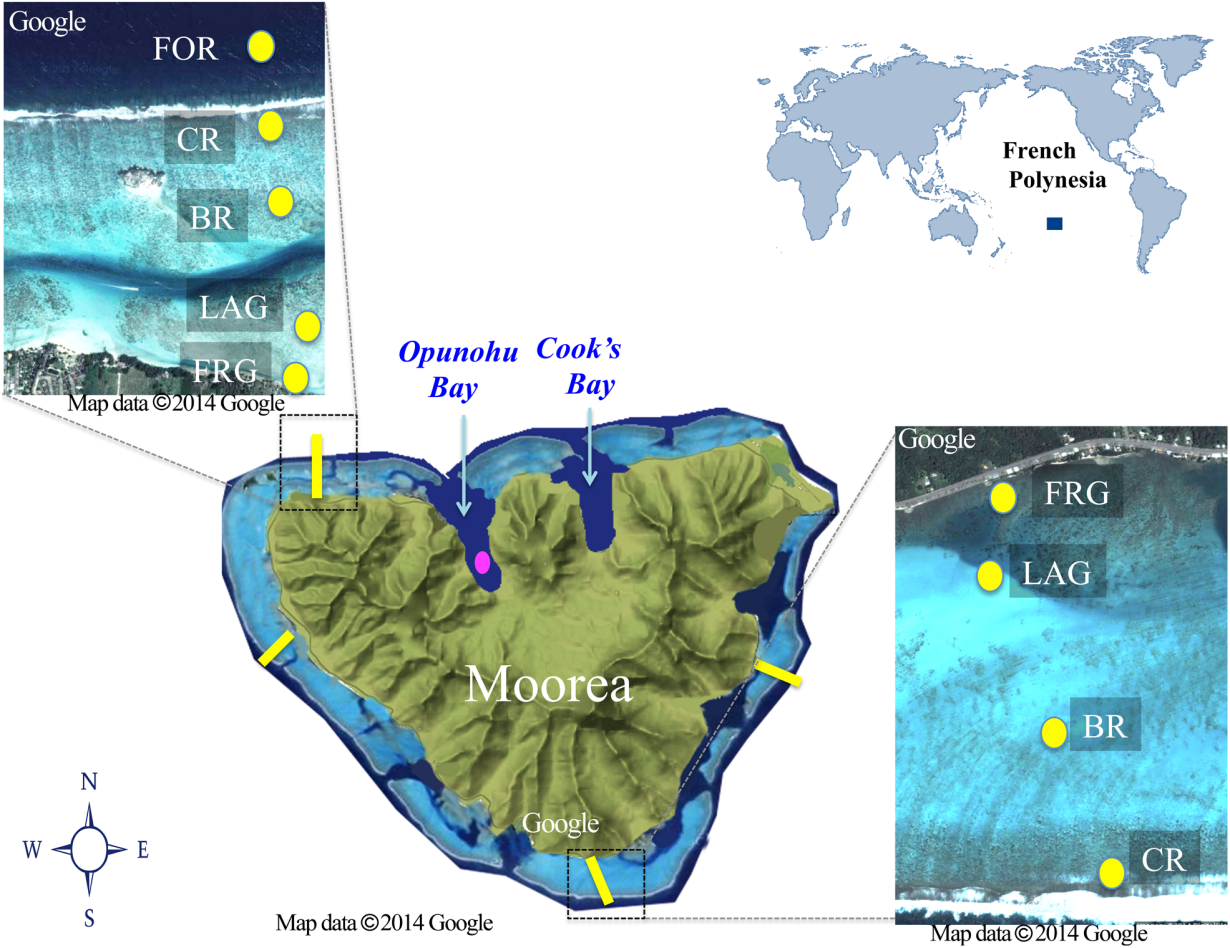
**Map of the study area.** Spatial samples were collected along four regional reef transects (east, west, south, and north; yellow lines). Each transect crossed distinct reef habitats: fringing reef (FGR), lagoon (LAG), back reef (BR), crest (CR), and fore reef (FOR); yellow dots. Note the FOR was sampled only on the north transect. Temporal samples were collected every 2–3 days for 21 days in a site in the Opunohu Bay (pink dot). Aerial views of the north and south transects are shown.

This study was conducted at Moorea Island, in French Polynesia, in the South Pacific Ocean (**Figure 1**), during the dry season in August 2013. Moorea is a high basaltic island surrounded by barrier reefs that extend between 500 and 1500 m offshore, creating semi-enclosed back reef lagoons (e.g., reviewed in Leichter et al., 2013; **Figure 1**). For consistency herein, the semi-enclosed lagoons of individual reef platforms will be referred to as “lagoon”. Eleven passes connect the lagoons to the open ocean, with some continuing near-shore as narrow deep channels (10–20 m width, 10–30 m depth). The typical reef zonation includes a fringing reef (FGR) nearest to shore (10–100 m width, <1 m depth), a shallow lagoon (100–1000 m width, 1–6 m depth) interrupted with the occasional along-shore channel, a back reef (100–200 m width, 1–3 m depth), a reef crest (10–50 m width, <1 m depth) and an oceanward fore reef on a high downslope (50–200 m width, 2–60 m depth). On the north shore, the lagoon is connected to two narrow, 3 km long straight water bays [Opunohu Bay (OB) and Cook’s Bay, <90 m deep]. Both bays are influenced by small river discharges that peak during rainfall; OB is also influenced by runoff from a nearby agricultural area that includes farming of prawns (Wolanski and Delesalle, 1995; Hench et al., 2008).

### SEAWATER SAMPLING

Two types of sampling were conducted to examine spatiotemporal variability in biotic (e.g., viruses, heterotrophic, and autotrophic microbes) and abiotic (e.g., nutrients, longitude, latitude) variables. For the spatial study, samples were collected separately along four cross-reef transects located in four different geographic regions (i.e., north, east, west, and south) surrounding Moorea. For each transect, seawater was collected at four different reef habitats starting offshore and moving toward inshore: reef crest (CR), back reef (BR), lagoon (LAG), and FGR (**Figure 1**). An additional site located in the fore reef (FOR) was also collected for the north transect (**Figure 1**). FOR sites from other transects could not be sampled due to logistical constraints. For the short-term temporal study, a site located in OB, was sampled every 2–3 days from 8 to 27 August 2013.

Seawater samples (0.5 l) for nutrient concentration, microbial and viral abundances (see below) were collected for all sites using high-density polyethylene bottles at ca. 0.5 m below the surface. Additional seawater samples (4 l) were collected for viral production (VP) assays (see below) using 4-l low-density polyethylene collapsible Cubitainers^®^. All bottles and cubitainers were acid washed (∼10% HCl), rinsed with MilliQ^®^ water and then rinsed several times with *in situ* seawater before collection. All samples were transported back to the onshore laboratory and processed in under 2 h following collection. Samples were consistently collected between 9:00 and 12:00 h to avoid diel variation.

### PREPARATION OF SAMPLES FOR FLOW CYTOMETRY

Duplicate aliquots (1.8 ml) of each sample were dispensed into 2 ml-cryotubes containing gluteraldehyde (0.5% final concentration, electron microscopy grade, Sigma-Aldrich). Samples were fixed at 4^∘^C for 30 min then immediately frozen at -80^∘^C, before being analyzed using FC within 4–6 weeks (see below). Samples were shipped frozen on dry-ice back to Oregon State University (OSU). Due to logistic constraints, the samples could not be flash-frozen in liquid nitrogen before being stored at -80^∘^C. This is known to account for some virus losses, however they are reported to be minimal (<10%; Brussaard, 2004a). We therefore expect that virus loss in our samples was minimal, and that our data represent conservative estimates of VA. FC analysis of viruses, heterotrophic bacteria and phytoplankton were performed on a Becton Dickinson (BD) FACSCalibur flow cytometer (15 mW argon laser exciting at 488 nm, BD, San Jose, CA, USA), as described below.

### ENUMERATION OF VIRUSES AND HETEROTROPHIC BACTERIA

**FIGURE 2:**
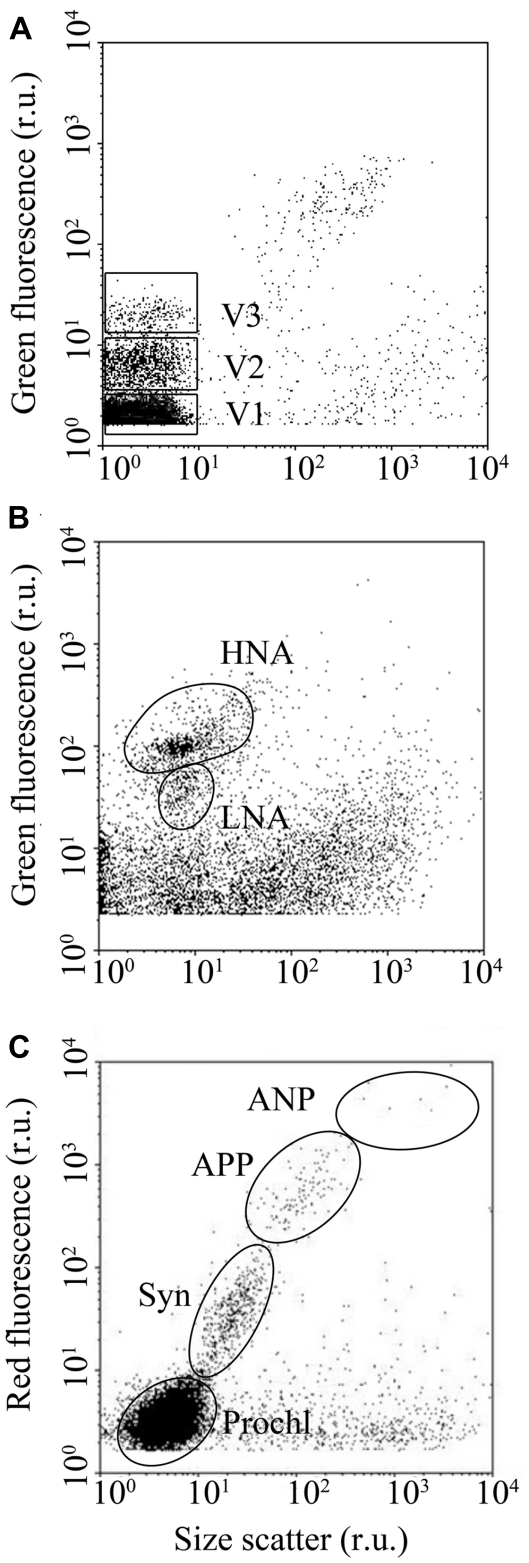
**Examples of typical flow cytograms of **(A)** viruses,**(B)** heterotrophic bacteria, and **(C)** phytoplankton.** Three viral subsets, with low, medium, and high nucleic acid fluorescence (V1, V2 and V3, respectively), two heterotrophic bacterial subsets with high and low nucleic acid fluorescence (HNA and LNA, respectively) and four phytoplankton subsets (*Prochl, Prochlorococcus*; *Syn, Synechococcus*; APP, autotrophic picoplankton; ANP, autotrophic nanoplankton) were distinguished using flow cytometry.

Viruses and heterotrophic bacteria were enumerated separately according to standard protocols outlined in Payet and Suttle (2008) and Brussaard et al. (2010), respectively. Viruses and heterotrophic bacteria were discriminated by their signals in a bivariate scatter plot of side scatter (SSC) vs. green fluorescence (FL1, 530/30 nm), using FL1 as the threshold trigger. At least three viral subgroups were discriminated based on their relative SYBR green I fluorescence (V1, V2, and V3, respectively; **Figure 2A**). The total VA (VA) presented in this study is the sum of V1, V2, and V3. FC allowed separation and enumeration of a high nucleic acid (HNA) containing bacteria and a low nucleic acid (LNA) containing bacteria on the basis of their SSC vs. FL1 signals (**Figure 2B**; Gasol et al., 1999; Lebaron et al., 2001). Total heterotrophic bacterial abundance (BA) was calculated as the sum of HNA and LNA cells.

### ENUMERATION OF PHYTOPLANKTON

Phytoplankton were enumerated using FC, following standard procedures (Olson et al., 1993; Marie et al., 2001). Just before the analysis, a mixture of yellow–green fluorescent 0.92 and 3 μm beads were added to the samples (ca. 10^5^ beads ml^-1^ final concentration) for internal standard. The threshold trigger was set to FL3. Phytoplankton populations were differentiated based on SSC, chlorophyll fluorescence (FL3) and phycoerithrin fluorescence (FL2, 585/42 nm) signals. In this study, FC differentiated autotrophic pico- (<2 μm) and nanoplankton (2–20 μm); hereafter referred to as autotrophic picoplankton and autotrophic nanoplankton (APP and ANP, respectively), as well as the picocyanobacteria *Synechococcus* and *Prochlorococcus* (hereafter referred to as *Syn* and *Prochl*, respectively; **Figure 2C**). Total phytoplankton abundance (PA) was calculated as the sum of APP, ANP, *Syn,* and *Prochl*.

### MEASUREMENTS OF AMBIENT SEAWATER ABIOTIC VARIABLES

Samples (90 ml) were filtered through combusted GF/C filters (Whatman GF/C, 25 mm diameter, 0.45 μm pore size) for nutrient analyses. Filters were held using acid-cleaned polycarbonate filter holders. Filter holders were attached directly to the outlet of acid-cleaned 60 cc syringes. For each sample, seawater filtrates were collected into duplicate acid-cleaned 30 ml HDPE bottles and stored upright at -80^∘^C until analysis at OSU within 2 months. Concentrations of dissolved inorganic nitrate plus nitrite (N+N), ammonium (NH_4_), and soluble reactive phosphorus (SRP) were measured using a hybrid air-segmented flow system consisting of a Technicon AutoAnalyzer II (SEAL Analytical Ltd., Milwaukee, WI, USA) and an Alpkem Rapid Flow Analyzer (Alpkem Series 300, Corp., Clackamas, OR, USA) following standard colorimetric protocols adapted from Gordon et al. (1993). In this study, we define dissolved inorganic nitrogen (DIN) as the sum of N+N and NH_4_ concentrations.

### MEASUREMENTS OF LYTIC VIRAL PRODUCTION AND ACTIVITY

Lytic VP assays were carried out using the 4 l seawater samples collected (see above) from the FRG, BR, and CR within each transect, as well as in the OB for all the sampling dates. We used the viral-reduction approach of Winget et al. (2005) adapted from Wilhelm et al. (2002). Briefly, 900 ml of seawater was filtered through 20 μm mesh-size Nitex^®^ screen to remove large particles. Filtered sample was then reduced to ca. 100 ml using a 0.22 μm pore-size polysulfone (PES) membrane tangential flow filter (TFF, GE Healthcare, Life Sciences). This process reduces particles <0.22 μm in diameter (i.e., most viruses infecting prokaryotes) while retaining particles ranging in size between 0.22 and 20 μm (i.e., pro- and eukaryotic microbes). The resulting retentate was subsequently washed with 900 ml of ultrafiltered (UF) seawater (<100 kDa cutoff, PES membrane TFF, GE Healthcare, Life Sciences) made from the same original seawater to further reduce viral-size particles. When ca. 100 ml of retentate remained the sample was brought back to its original volume to produce a virus-reduced sample (i.e., 900 ml). All the TFF cartridges and tubing were cleaned with NaOH 0.1N and thoroughly rinsed with MilliQ^®^ water and UF seawater before use. On average, the viral-reduction approach removed 65 ± 5% (range: 22–92%) of *in situ* VA and 57 ± 9% (range: 16–81%) of *in situ* BA, respectively. The same flow rates and processing times were used in all experiments.

The resulting virus-reduced sample was dispensed into triplicate sterile 50 ml conical tubes (BD Flacon) before incubation at *in situ* temperature (26 ± 1^∘^C) for 12–18 h in a temperature-controlled room in the dark. Samples (1 ml) for determination of VA and BA were collected every 3–4 h. For each individual incubation, VP was estimated from the slope of a least-square linear regression fitted to VA increases over time after correcting for *in situ* BA losses during the filtration, as described in Wilhelm et al. (2002).

Viral turnover rates (VT, d^-1^), viral-induced mortality of bacteria (VMB, bacteria l^-1^ d^-1^), percentage of bacterial standing stock removed (%BSSr, d^-1^) and extracellular dissolved organic carbon released (OCr, μg C l^-1^ d^-1^) were calculated as in Wilhelm et al. (2002) and Payet and Suttle (2013; **Table 1**). We used a burst size (BS) of 30 viruses per lytic event, which was close to the average BS estimates of 28 reported in South Pacific tropical waters (Bouvy et al., 2012) and of 24 reported for marine environments (Parada et al., 2006). A cellular carbon quota of 20 fg C per marine bacterium was used to convert BA into organic carbon units (Lee and Fuhrman, 1987).

**Table 1:** Equations used in estimating viral production (VP), viral turnover (VT), viral-mediated mortality of bacteria (VMB), amount of organic carbon released upon viral lysis (OCr) and percent of bacterial standing stock removed due to viral lysis (BSSr).

Variables	Equations
VP (viruses L^-1^ d^-1^)	[VAtf – VAto/tf]x (BAa/BAto)
VT (d^-1^)	VP/VA
VMB (bacteria L^-1^ d^-1^)	VP/BS
OCr (μg C L^-1^ d^-1^)	VMB × 20 fg C cell^-1^
BSSr (% d^-1^)	100 × (VMB/BAa)

*VA, viral abundance; to, initial time point of incubation; tf, final time point of incubation; BA, bacterial abundance; BAa, ambient bacterial abundance; BS, burst size.*

### DATA ANALYSIS AND STATISTICS

Differences among mean biotic/abiotic variables during time-series and across reef-transects were tested by Kruskal–Wallis (KW) analysis of variance on ranks, as the data did not meet the assumptions of normal distribution and homoscedasticity needed for analysis of variance tests (Zar, 1999). When KW tests were significant, the Dunn’s *post hoc* test was performed to evaluate within group differences.

The distance-based linear model (DistLM) analysis (Legendre and Anderson, 1999; Anderson, 2004) was carried out to examine which biotic or abiotic variables were potential predictors of spatiotemporal variations in the viral variables (i.e., VA, V1, V2, V3, and VP). For this analysis, Bray–Curtis dissimilarity matrices of log-transformed data for a selected viral variable were fitted against the abiotic (see below) and biotic (i.e., log-transformed BA, HNA, LNA, PA, *Prochl*, *Syn*, ANP, and APP) variables. A forward selection procedure based on Akaike’s Information Criterion with a second-order bias correction for small sample size (AICc) measures of fit was used to determine which explanatory variables could best predict selected viral variables (see Burnham and Anderson, 2004). Highly correlated explanatory variables (*r* > 0.9) were omitted for the DistLM procedure. *P*-values were obtained using 999 random permutations of the data. For the spatial dataset, the abiotic variables included nutrient concentrations (log-transformed DIN and SRP) and coordinates (latitude and longitude). For the temporal dataset, the abiotic variables included log-transformed nutrients and time (number of days after first sampling). All the abiotic variables were normalized prior to DistLM procedure. Statistical analyses were performed using RStudio Version 0.97.551 (; Racine, 2012) and PRIMER 6 with the PERMANOVA+ add-on (PRIMER-E, Plymouth, UK; Clarke and Gorley, 2006; Anderson et al., 2008). Means ± standard deviations (SD) are reported in the text for specific data sets.

## RESULTS AND DISCUSSION

This study examined spatial and short-term temporal variability of VA and lytic activity in relation to changes in microbial population abundances and environmental conditions, for the first time, in coral reefs surrounding Moorea Island, in the South Pacific Ocean. The results show high spatial heterogeneity and relatively low temporal changes in VA and lytic activity, concomitant with shifts in microbial host population dynamics. Overall, our data suggest that viral-induced lysis can exert strong controlling influences on heterotrophic BA, with implications for nutrient and carbon fluxes in these oligotrophic ecosystems.

### ENVIRONMENTAL CONDITIONS

Relatively low mean nutrient concentrations measured at all sites confirmed the oligotrophic nature of this reef ecosystem, with SRP and DIN averaging 0.35 ± 0.08 μM and 0.49 ± 0.24 μM, respectively (data not shown). Although no significant differences in nutrient concentrations were detected among reef habitats, DIN concentrations were higher in the FRG (0.48 ± 0.14 μM) relative to the LAG (0.40 ± 0.13 μM) and BR (0.38 ± 0.09 μM), CR (0.34 ± 0.09 μM), and FOR (0.41 ± 0.10 μM; KW, *p* > 0.05). In the OB, DIN concentrations (0.93 ± 0.15 μM) were ∼1.9-fold higher, but not significantly different, than in the FRG (KW, *p* > 0.05). During the time-series, DIN concentrations remained relatively stable in the OB with low date-to-date variations (range: 0.7- to 1.2-fold). No significant differences were detected between sampling dates (KW, *p* > 0.05).

### HETEROTROPHIC BACTERIA DISPLAY SPATIAL VARIABILITY AND SHORT-TERM TEMPORAL STABILITY

**FIGURE 3:**
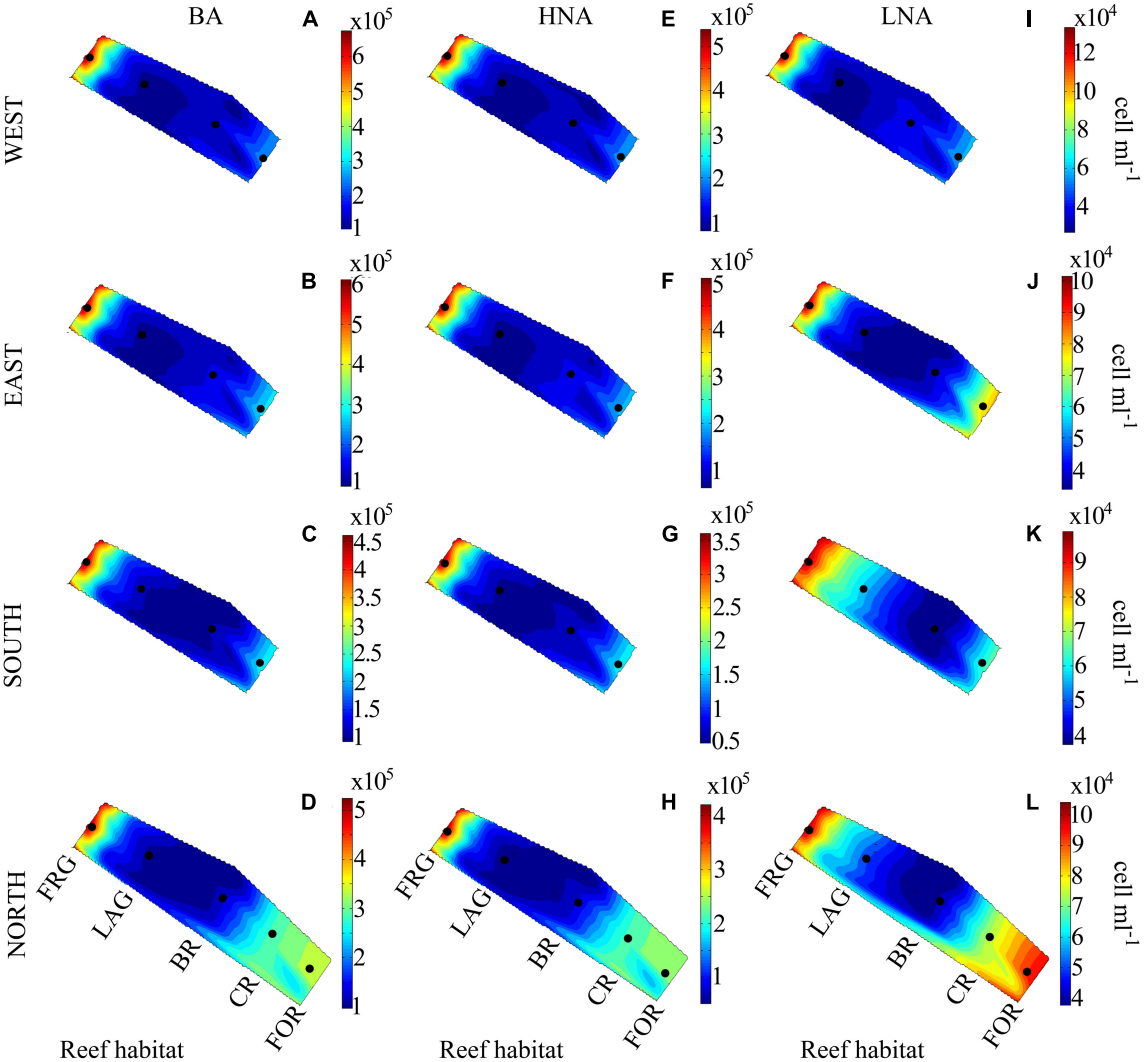
**Spatial distribution of heterotrophic bacteria along four regional transects (east, west, south, and north) surrounding Moorea. (A–D)** Bacterial abundance (BA), **(E–H)** high nucleic acid (HNA) bacterial abundance and **(I–L)** low nucleic acid (LNA) bacterial abundance. Each transect crossed distinct reef habitats: the fringing reef (FRG), lagoon (LAG), back reef (BR), crest (CR), and fore reef (FOR). Note the FOR was sampled only on the north transect. Black dots indicate the relative position of the samples collected. Contour plots indicate the mean values of duplicate samples.

**FIGURE 4:**
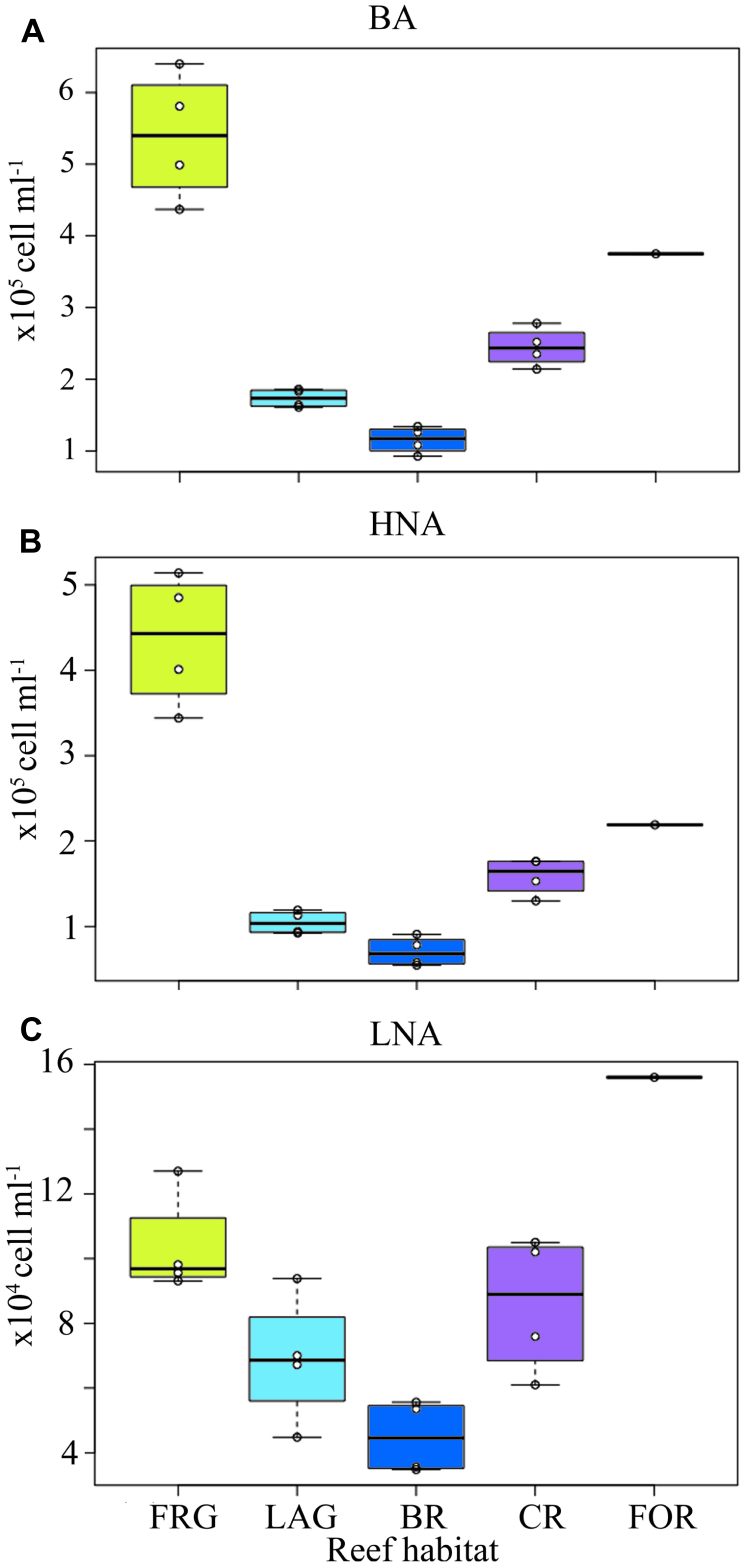
**Spatial distribution of heterotrophic bacteria across the fringing reef (FRG), lagoon (LAG), back reef (BR), crest (CR), and fore reef (FOR) habitats. (A)** Heterotrophic bacterial abundance (BA), **(B)** high nucleic acid (HNA) bacterial abundance and **(C)** low nucleic acid (LNA) bacterial abundance. Note the FOR was sampled only once on the north transect. Boxplots represent 50th (median), 75th and 25th percentiles. Circles represent mean values of duplicate samples collected for each transect.

Bacterial abundance averaged 2.7 × 10^5^ ± 1.5 × 10^5^ cells ml^-1^ and did not vary significantly among the four transects (KW, *p* > 0.05; **Figures 3A–D**). However, consistent spatial trends emerged within transects, according to reef habitat (**Figure 4A**). On average, BA decreased 1.5-fold from the FOR toward the CR and a ∼2-fold from the CR toward the BR and LAG. Similar decreasing trends in BA from the FOR toward the BR were reported during a long-term study in Moorea (Nelson et al., 2011). The authors hypothesized that low wave-driven circulation and long water turnover time in the BR could increase encounter rates between bacteria and heterotrophic benthic organisms, resulting in low abundances. BA increased ∼3-fold in the FRG relative to the BR and LAG (KW with Dunn’s test, *p* < 0.05; **Figure 4A**). Given that DIN concentrations were ∼1.2-fold higher in the FRG relative to the LAG and BR, it is likely that some micro-gradients in nutrient availability may have occurred in the FRG. For instance, small inputs from terrestrial runoff may have increased nutrient availability, which in turn stimulated heterotrophic BA. For example, Weinbauer et al. (2010a) reported increases of heterotrophic BA in response to small increases in nutrient availability due to terrestrial runoffs in another reef ecosystem. It is also likely that organic matter releases from benthic organisms may have caused micro-gradients in nutrient and carbon availability that stimulated ambient heterotrophic bacteria in the FRG. This is consistent with previous findings (Kline et al., 2006; Haas and Wild, 2010; Haas et al., 2011) that coral and macroalgal exudates of organic matter can enhance heterotrophic microbial abundance and activity, with implications for community structure (Haas et al., 2011, 2013; Nelson et al., 2013).

Consistent with BA, LNA and HNA containing cells followed decreasing trends along transects () and averaged 1.9 × 10^5^ ± 1.4 × 10^5^ and 9.2 × 10^4^ ± 4.2 × 10^4^ cells ml^-1^, respectively. HNA cells were more abundant than LNA cells (*t*-test on ranks, *p* < 0.01) and contributed 65 ± 9% of total BA along all transects. The proportion of HNA cells was higher in the FRG relative to the LAG and BR (KW with Dunn’s test, *p* < 0.05), contributing up to 81% of total BA. In contrast, the proportion of LNA cells increased oceanward (KW, *p* >0.05) and contributed up to 44% of total BA in the FOR. Seymour et al. (2005) reported similar increased proportions of HNA cells in proximity to corals. HNA cells are reported to be large contributors to heterotrophic microbial activities, particularly in nutrient-replete conditions (Gasol et al., 1999; Lebaron et al., 2001; Servais et al., 2003). Therefore these results may indicate increased heterotrophic microbial activity in the FRG. However, recent surveys also have shown LNA cells, which are members of the abundant alphaproteobacterial clade SAR11, can be highly active, particularly in nutrient-depleted conditions (Zubkov et al., 2001; Jochem et al., 2004; Longnecker et al., 2005; Mary et al., 2008; Hill et al., 2010; Gomez-Pereira et al., 2013).

**FIGURE 5:**
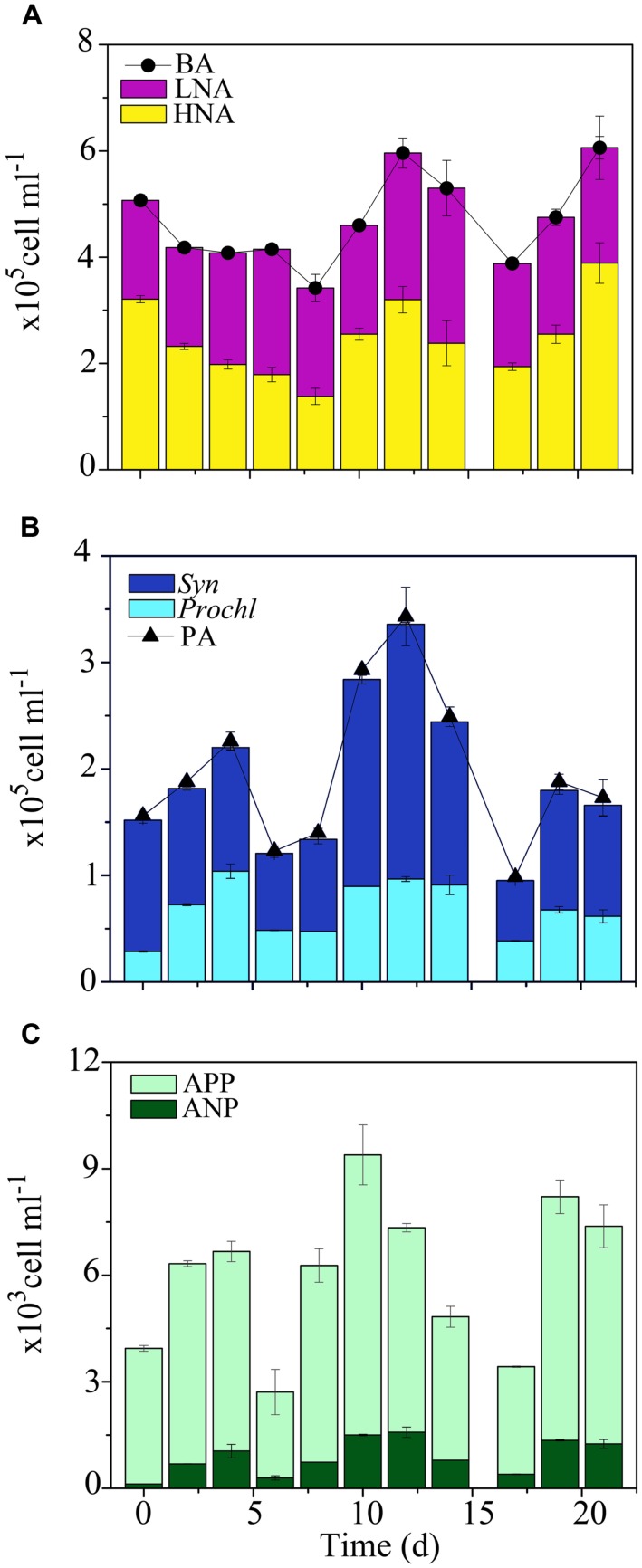
**Short-term temporal distribution of autotrophic and heterotrophic microbes in the Oponuhu Bay. (A)** Heterotrophic bacterial abundance (BA), high nucleic acid (HNA) bacterial abundance and low nucleic acid (LNA) bacterial abundance. **(B)** Phytoplankton abundance (PA), *Prochlorococcus* (*Prochl*), and *Synechococcus* (*Syn*) abundances. **(C)** Autotrophic pico- and nanoplankton (APP and ANP, respectively) abundances.

In the OB, BA averaged 5.6 × 10^5^ ± 0.9 × 10^5^ cells ml^-1^ and was significantly higher than sites along the transects (KW, *p* < 0.05; **Figure 5A**). HNA cells outnumbered LNA cells in the OB, representing 67 ± 8% of the total BA. This higher heterotrophic microbial abundance, and presumably activity, parallel the general pattern observed in the adjacent Cook’s Bay (Nelson et al., 2011), that has relatively similar hydrological settings. In the OB, there were notable increases in suspended organic matter in the water column, as evidenced by reduced visibility (<5 m). Near-bottom currents that continuously re-suspend silty bottom of the bay, concomitant with small terrigenous inputs from a river near the tip of the bay, may explain such increases in suspended particles, as was reported previously in the OB and Cook’s Bay (Wolanski and Delesalle, 1995; Hench et al., 2008; Nelson et al., 2011). Thus, potential nutrient supply in the form of suspended organic matter may have been important in sustaining high heterotrophic microbial abundance in the OB. The time-series in the OB revealed small temporal oscillations in BA with low date-to-date changes (range: 0.7- to 1.5-fold) and relatively stable proportions of HNA cells (KW, *p* > 0.05; **Figure 5A**), suggesting relative homogeneity in the abundance structure of the heterotrophic microbial communities.

### AUTOTROPHIC MICROBES DISPLAY SPATIAL VARIABILITY AND SHORT-TERM TEMPORAL STABILITY

**FIGURE 6:**
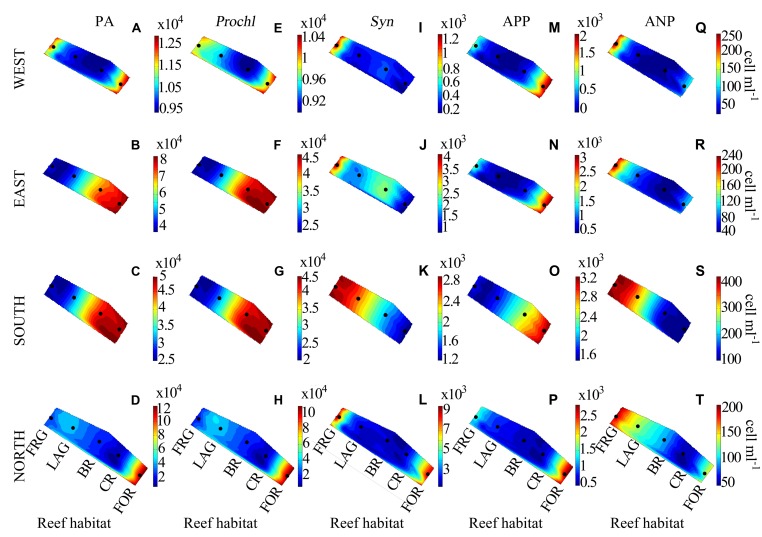
**Spatial distribution of autotrophic microbes along four regional transects (east, west, south, and north) surrounding Moorea.(A–D)** Phytoplankton abundance (PA), **(E–H)** *Prochlorococcus* (*Prochl*), **(I–L)** *Synecochococcus* (*Syn*), **(M–P)** autotrophic picoplankton (APP), and **(Q–T)** autotrophic nanoplankton (ANP) abundances. See **Figure 3** for legend.

**FIGURE 7:**
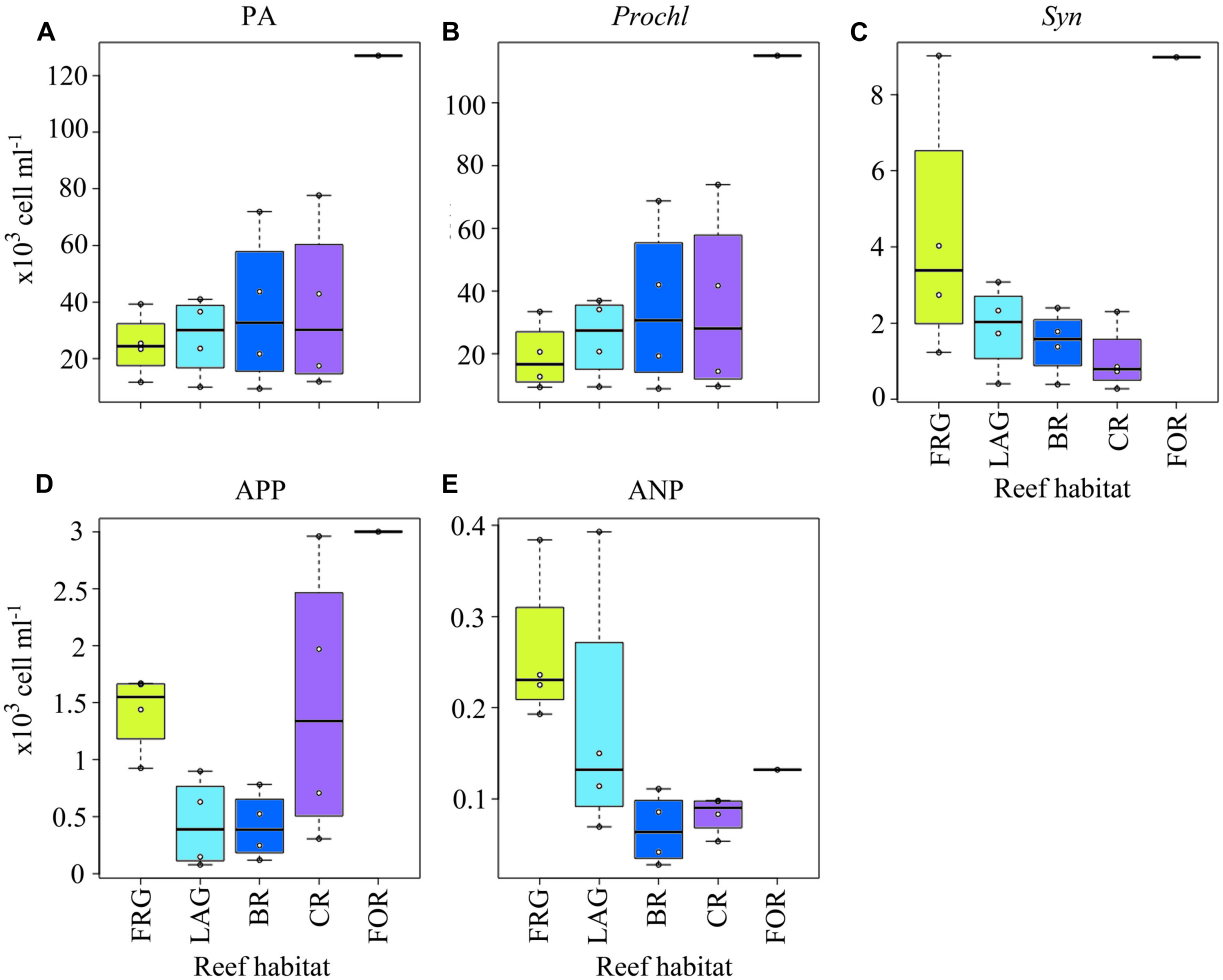
**Spatial distribution of phytoplankton across the fringing reef (FRG), lagoon (LAG), back reef (BR), crest (CR), and fore reef (FOR) habitats. (A)** Phytoplankton abundance (PA), **(B)** *Prochlorococcus* (*Prochl*) abundance, **(C)** *Synechococcus* (*Syn*) abundance, **(D)** autotrophic picoplankton (APP) abundance, and **(E)** autotrophic nanoplankton (ANP) abundance. See **Figure 4** for legend.

For the transects PA averaged 3.6 × 10^4^ ± 3.0 × 10^4^ cells ml^-1^ and displayed consistent spatial trends (**Figures 6A–D**). Although PA gradually decreased ∼5-fold from the FOR toward the FRG (**Figure 7A**), no significant differences were detected among reef habitats (KW, *p* > 0.05). Similar to BA, there were dynamic spatial shifts in the phytoplankton community, with *Prochl* cells (3.4 × 10^4^ ± 2.9 × 10^4^ cells ml^-1^) dominating total PA relative to *Syn* (2.6 × 10^3^ ± 2.6 × 10^3^ cells ml^-1^), APP (1.1 × 10^3^ ± 0.9 × 10^3^ cells ml^-1^) and nanoplankton (ANP; 142 ± 108 cells ml^-1^) cells. All phytoplankton subsets except ANP cells followed decreasing trends from the FOR toward the CR and BR (**Figures 6E–T**), likely due to increased encounter rates with benthic filter feeders, as mentioned above. In the FRG, the dominant *Prochl* subset continuously decreased while the abundance of *Syn*, ANP, and APP cells markedly increased (**Figures 7B–E**). Organic matter and nutrient supply from benthic exudates may have stimulated microalgae with higher nutrient requirements in the FRG. However it is noteworthy that *Prochl*, which are known to cope better than *Syn* and eukaryotic phytoplankton in nutrient-depleted seawater (e.g., reviewed in Scanlan et al., 2009), were still prevailing in the FRG. This suggests that the nutrient supply was not sufficient to shift the phytoplankton community from *Prochl*-dominated toward *Syn*-dominated communities, as has been reported in other coastal tropical reef waters (e.g., reviewed in Charpy et al., 2012).

Similar to BA, higher overall mean PA was measured in the OB (2.2 × 10^5^ ± 1.1 × 10^5^ cells ml^-1^; KW, *p* < 0.05). In contrast to the transects, *Syn* was the most abundant phytoplankton, followed by *Prochl* (58 ± 4% and 39 ± 5%, respectively; **Figures 5B,C**). This shift in autotrophic microbial community structure and abundance supports the hypothesis that suspended organic matter and associated nutrients may have stimulated autotrophic cells with high nutrient demand. During the time-series in the OB, PA displayed similar temporal oscillations to BA with relatively low date-to-date changes (range: 0.4- to 2.5-fold; **Figure 5B**; KW, *p* > 0.05). Phytoplankton subsets also remained relatively unchanged (KW, *p* > 0.05), indicating a relatively stable autotrophic microbial community.

### VIRUSES DISPLAY SPATIAL VARIABILITY AND SHORT-TERM TEMPORAL STABILITY DRIVEN BY BIOTIC AND ABIOTIC FACTORS

**FIGURE 8:**
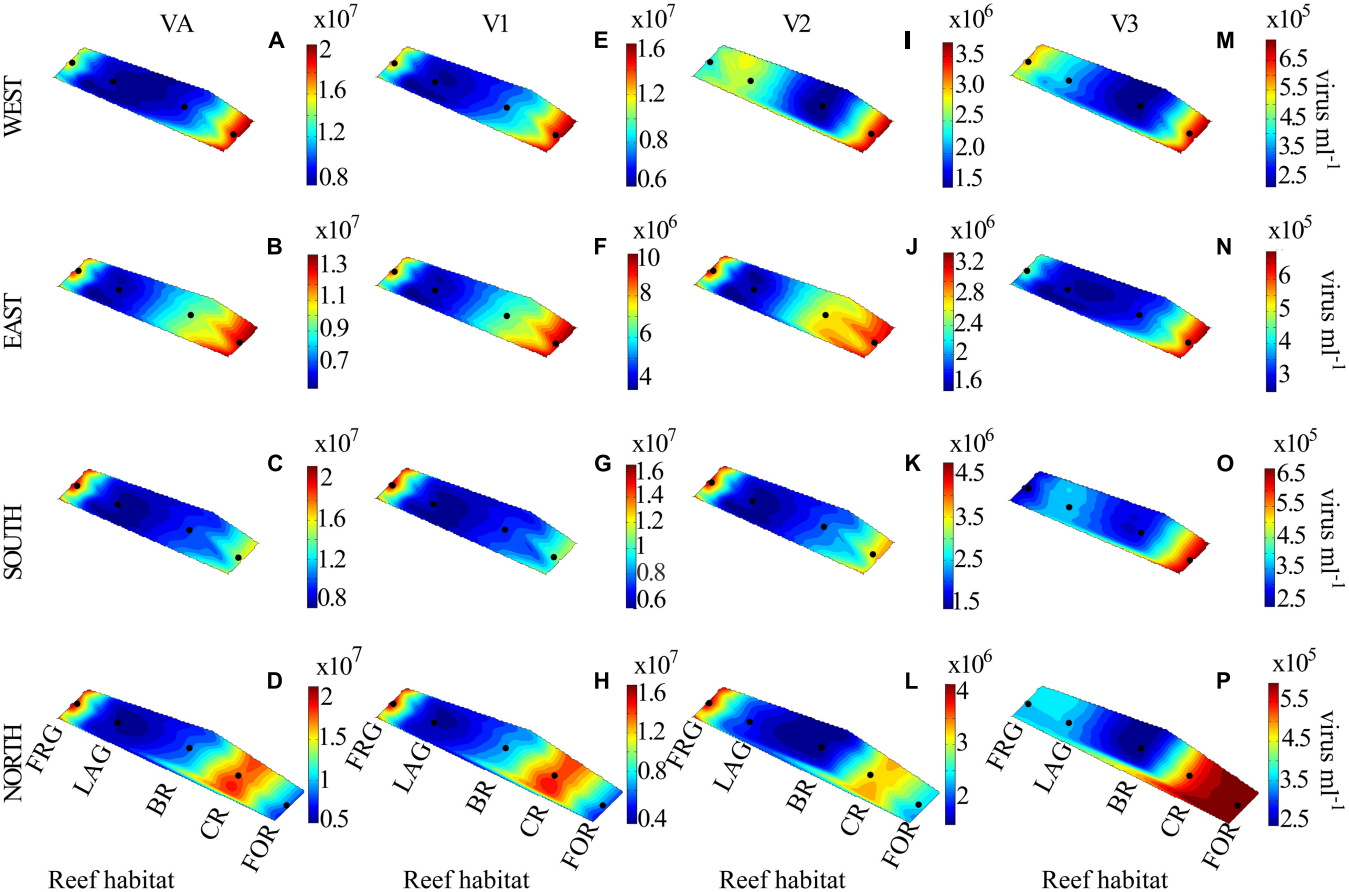
**Spatial distribution of viruses along four regional transects (east, west, south, and north) surrounding Moorea. (A–D)** viral abundance (VA), **(E–H)** low nucleic acid viral subset abundance (V1), **(I–L)** medium nucleic acid viral subset abundance (V2), and **(M–P)** high nucleic acid viral subset abundance (V3). See **Figure 3** for legend.

**FIGURE 9:**
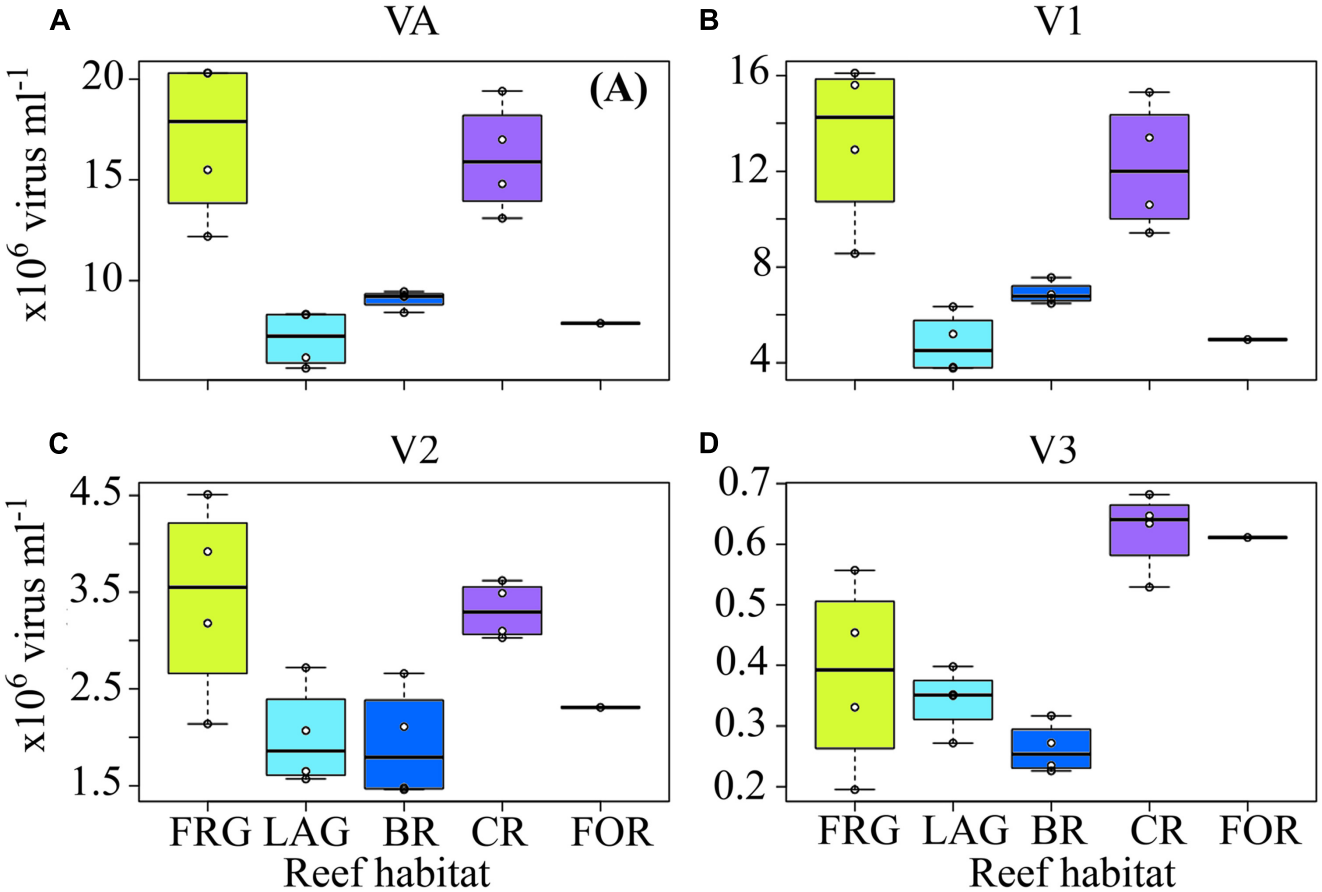
**Spatial distribution of viruses across the fringing reef (FRG), lagoon (LAG), back reef (BR), crest (CR), and fore reef (FOR) habitats. (A)** Viral abundance (VA), **(B)** low nucleic acid viral subset abundance (V1), **(C)** medium nucleic acid viral subset abundance (V2), and **(D)** high nucleic acid viral subset abundance (V3). See **Figure 4** for legend.

Along cross-reef transects, VA averaged 1.2 × 10^7^ ± 0.5 × 10^7^ viruses ml^-1^ and was within ranges previously reported for coral reef waters (Paul et al., 1993; Seymour et al., 2005; Patten et al., 2006; Bouvy et al., 2012). Viral abundance followed similar spatial trends within the four transects (**Figures 8A–D**), with lowest values in the BR and LAG (**Figure 9A**). On average, VA was ∼2.2- to 2.4-fold higher in the FRG and CR, relative to the BR and LAG (**Figure 9A**). DistLM for VA vs. biotic (i.e., BA and PA) and abiotic (i.e., DIN, SRP, longitude and latitude) variables indicated that these variables contributed to 24 and 38% of spatial variability in VA, respectively (**Table 2**). Among biotic variables, BA was the best predictor of VA, while SRP and DIN were the best abiotic predictors (**Table 2**). Interestingly, these results suggest that microbial host abundance only partially explained spatial VA distribution along transects, and that other unmeasured ecological processes may have influenced the distribution of VA. For example, small-scale changes in hydrological conditions may have influenced host distribution and metabolic activities, with implications for host-virus dynamics and subsequent spatial patterns of VAs. Alternatively, increases in VT time relative to host microbial abundance may have dampened the relationship between viruses and host microbes.

**Table 2:** Results of separate distance-based linear model (DistLM), with forward procedure, fitting viral abundance (VA), low nucleic acid viral V1 subset abundance, medium nucleic acid viral V2 subset abundance, high nucleic acid viral V3 subset abundance and viral production (VP) against biotic and abiotic variables.

Response variables	Factors	Predictors	Pseudo-*F*	*p*	Proportional	Cumulative
*Spatial*						
VA	Biotic	BA	4.82	**0.04**	0.24	0.24
	Abiotic	SRP	9.15	**0.01**	0.38	0.38
		DIN	3.74	0.06	0.13	0.51
V1	Biotic	*Prochl*	3.36	0.06	0.18	0.18
		LNA	4.65	**0**.**04**	0.21	0.39
	Abiotic	SRP	7.35	**0.01**	0.33	0.33
		DIN	3.76	0.06	0.14	0.47
V2	Biotic	HNA	5.59	**0.03**	0.27	0.27
	Abiotic	SRP	7.87	**0.02**	0.34	0.34
V3	Biotic	APP	8.69	**0.02**	0.38	0.38
	Abiotic	SRP	2.19	0.17	0.13	0.13
VP	Biotic	BA	3.81	0.06	0.28	0.28
	Abiotic	SRP	2.19	0.17	0.18	0.18
*Temporal*						
VA	Biotic	PA	3.63	0.08	0.32	0.32
	Abiotic	SRP	6.43	**0.04**	0.45	0.45
V1	Biotic	*Syn*	2.30	0.09	0.21	0.21
	Abiotic	DIP	5.03	0.06	0.39	0.39
V2	Biotic	LNA	4.30	0.07	0.35	0.35
	Abiotic	SRP	8.18	**0.03**	0.51	0.51
V3	Biotic	APP	3.04	0.10	0.22	0.22
		*Syn*	4.01	**0.05**	0.29	0.50
	Abiotic	SRP	6.18	**0.04**	0.44	0.44
VP	Biotic	PA	3.54	0.09	0.28	0.28
	Abiotic	SRP	8.85	**0.02**	0.50	0.50
		DIN	10.41	**0.01**	0.28	0.78

*The analyses were conducted separately for biotic and abiotic variables as well as for samples collected along four cross-reef transects and during the time-series in the Oponuhu Bay. Pseudo-F and p-values were obtained by permutations (n = 999). P-values at a significance level of 0.05 are in **bold**. Proportional and cumulative percentages of variance are also reported. BA, bacterial abundance; LNA, low nucleic acid bacteria; HNA, high nucleic acid bacteria; SRP, soluble reactive phosphorus; DIN, dissolved inorganic nitrogen; PA, phytoplankton abundance; APP, autotrophic picoplankton; Syn, Synechococcus; Prochl, Prochlorococcus.*

**FIGURE 10:**
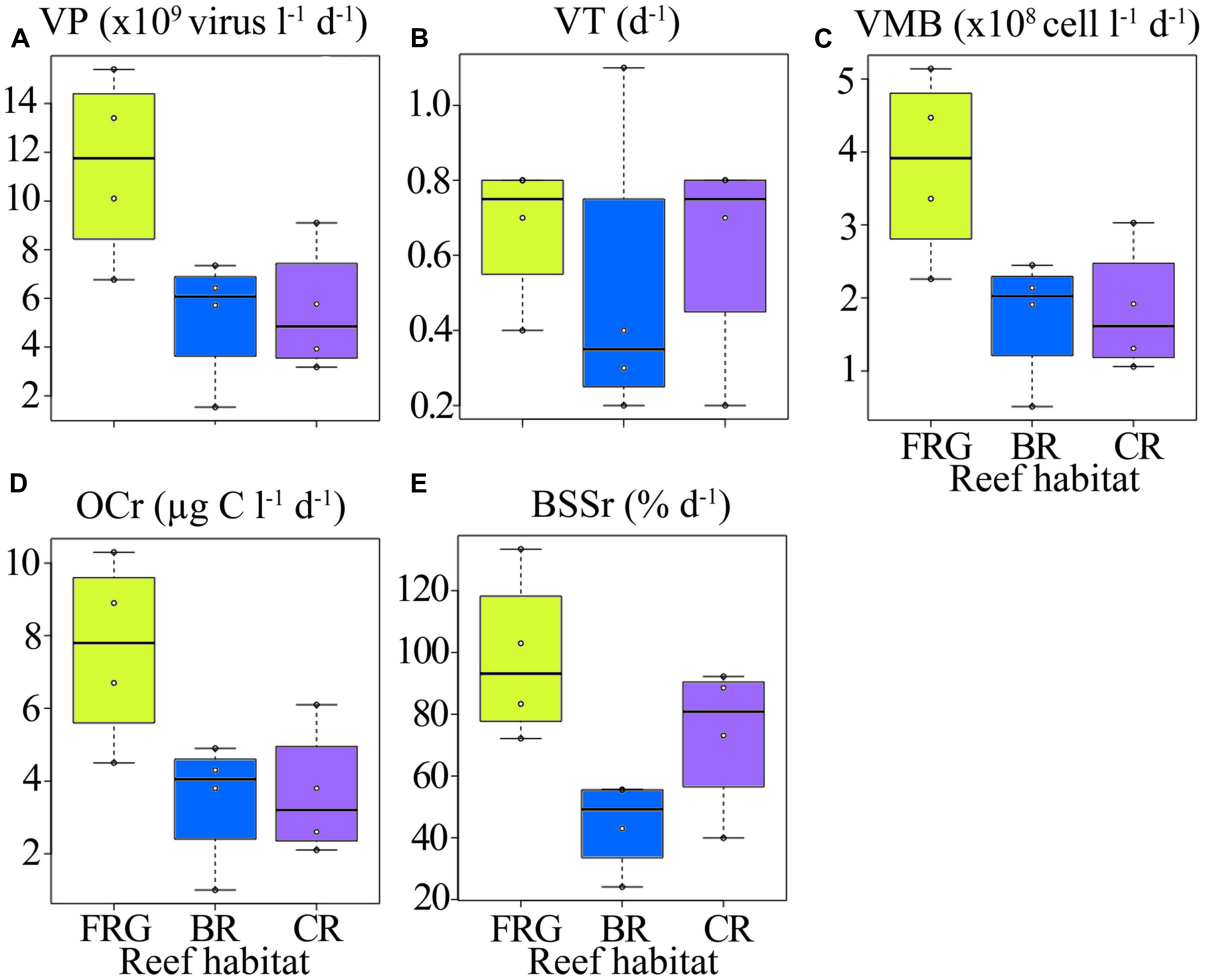
**Spatial distribution of viral lytic variables across the fringing reef (FRG), lagoon (LAG), back reef (BR), crest (CR), and fore reef (FOR) habitats. (A)** Viral production (VP), **(B)** viral turnover (VT), **(C)** viral-mediated mortality of bacteria (VMB), **(D)** amount of organic carbon released upon viral lysis (OCr), and **(E)** proportion of bacterial standing stock removed due to viral lysis (BSSr). See **Figure 4** for legend.

Higher VA was measured in the OB relative to other reef sites, with an overall mean value of 1.9 × 10^7^ ± 0.9 × 10^7^ viruses ml^-1^. During the time-series, VA displayed relatively similar temporal oscillations to BA and PA, with relatively low date-to-date changes (range: 0.3- to 2.4-fold; **Figure 10A**; KW, *p* > 0.05). DistLM for temporal VA vs. biotic (i.e., BA and PA) and abiotic (i.e., SRP, DIN and time) variables indicated that these variables explained 32 and 45% of temporal variability, with PA and SRP as their main predictors (**Table 2**). As outlined in the above section, the relatively weak relationships among VA, the measured biotic and abiotic variables suggest that other unmeasured variable(s) may be partially responsible for the observed temporal variability in virus–host dynamics, with implications for temporal distributions of VA.

### SPATIOTEMPORAL DISTRIBUTION OF VIRAL SUBSETS INDICATES DYNAMIC VIRAL ASSEMBLAGES

Based on their fluorescence properties, FC analysis revealed at least three viral subsets (i.e., V1, V2, and V3), with V1 and V2 contributing to most of the VA (70 and 25%, respectively). This is consistent with subsets reported in other marine ecosystems (Baudoux et al., 2007; Evans et al., 2009; Brussaard et al., 2010; Mojica et al., 2014), however it should be noted that in some environments, only V1 and V2 are readily detected (Patten et al., 2006; Seymour et al., 2006; Payet and Suttle, 2008).

Along transects, V1 and V3 displayed relatively high spatial variability (**Figures 8E–H, M–P**), with significant increases in V1 in the FRG relative to the LAG and significant increases in V3 in the CR relative to the BR (KW with Dunn’s test, *p <* 0.05; **Figures 9B,D**). V2 displayed relatively low spatial variability along transects (**Figures 8I–L**), with no significant differences detected among reef sites (KW, *p >* 0.05; **Figure 9C**).

Distance-based linear model for spatial V1, V2, and V3 abundances indicated significant relationships with biotic (i.e., HNA, LNA, *Syn*, *Prochl*, APP, and ANP) and abiotic (i.e., DIN, SRP and coordinates) variables (**Table 2**). Biotic and abiotic variables explained 39 and 47% of spatial variability in V1, respectively, with both LNA and *Prochl,* and both SRP and DIN as the best predictors (**Table 2**). Previous studies have reported that LNA cells contain a larger proportion of small bacteria from the alphaproteobacterial clade SAR11; these SAR11 bacteria typically co-occur with *Prochl* in nutrient-depleted waters (Hill et al., 2010; Gomez-Pereira et al., 2013). Therefore it may be that spatial patterns in V1 abundances were more associated with changes in these autotrophic and heterotrophic bacterial subsets across the reef. Biotic and abiotic variables explained 27 and 34% of spatial variability in V2, with HNA and SRP as the best predictors (**Table 2**). This suggests that viruses in the V2 subset were associated with heterotrophic microbes, with presumably high metabolic activity as outlined above. Biotic and abiotic variables explained 50 and 44% of the temporal variability in V3, respectively, with *Syn*, APP, and SRP as the best predictors (**Table 2**). This indicates that V3 is comprised of viruses that are associated with autotrophic microbial host cells.

Similar to temporal patterns in microbial community structure, proportions of V1, V2, and V3 remained relatively stable during the time-series in the OB, with only small date-to-date changes (**Figure 10A**). This suggests that viral community structure was relatively homogeneous over time. DistLM for temporal V1, V2, and V3 abundances revealed significant relationships with biotic and abiotic variables in the OB (**Table 2**). Similar to temporal variations in overall VA, associations between viral subsets and biotic/abiotic variables tended to be stronger than those observed for transects. For V1, biotic and abiotic variables explained 21 and 39% of temporal variability, respectively, with *Syn* cells and SRP as the main predictors (**Table 2**). For V2, biotic and abiotic variables explained 35 and 51% of temporal variability, with LNA cells and SRP as the main predictors (**Table 2**). For V3, biotic and abiotic variables explained 50 and 44% of temporal variability in V3 abundances, respectively, with *Syn,* APP and SRP as the best predictors (**Table 2**). Different best predictors in the OB indicate that V1, V2, and V3 subsets may be influenced by different ecological factors than those within transects. However, V3 subset had similar predictors in both the OB and transects, suggesting these viruses are associated with changes in autotrophic microbial communities.

### SPATIOTEMPORAL DISTRIBUTION OF LYTIC ACTIVITY SUGGESTS VIRUSES IMPACT MICROBIAL MORTALITY AND CARBON CYCLING

**FIGURE 11:**
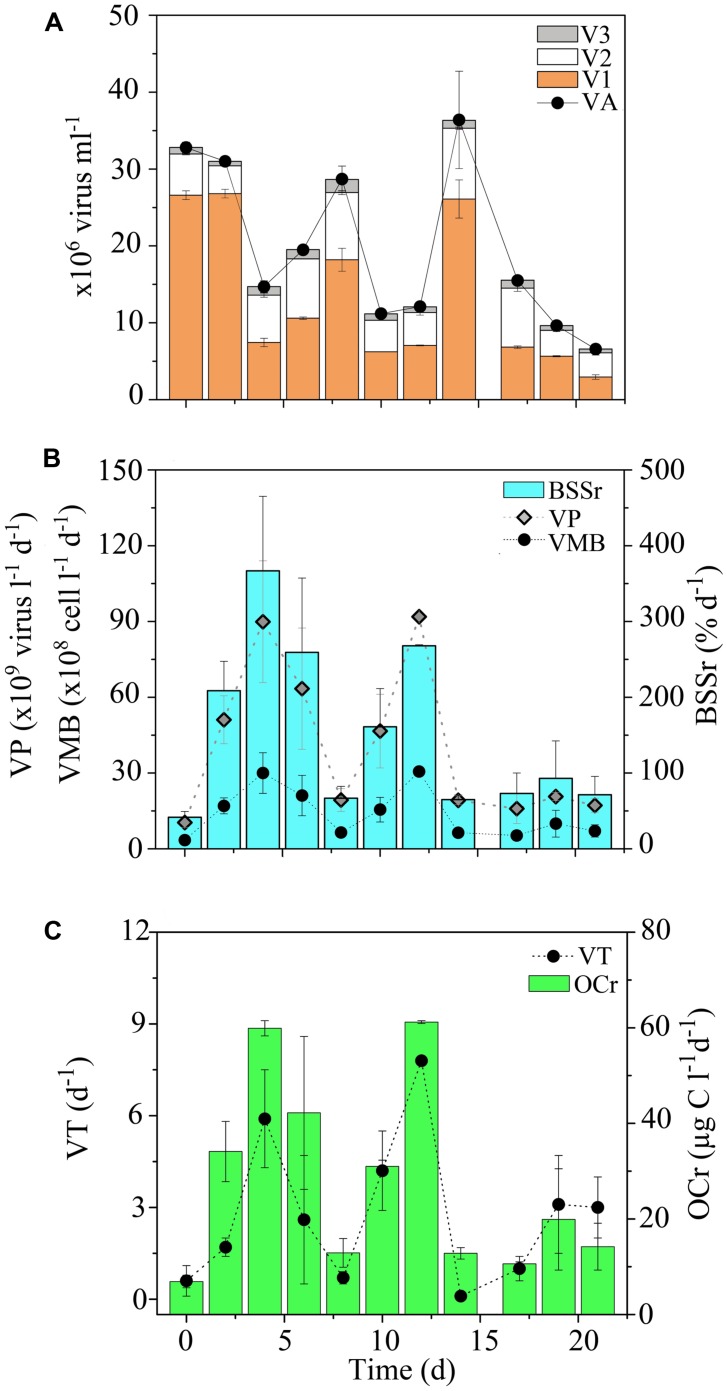
**Short-term temporal distribution of viruses in the Opunohu Bay. (A)** Viral abundance (VA), low nucleic acid viral subset abundance (V1), medium nucleic acid viral subset abundance (V2) and high nucleic acid viral subset abundance (V3), **(B)** viral production (VP), viral-mediated mortality of bacteria (VMB) and proportion of bacterial standing stock removed due to viral lysis (BSSr), and **(C)** amount of organic carbon released upon viral lysis (OCr) and viral turnover (VT) in the Oponuhu Bay. Error bars represent standard deviations.

Along transects, estimates of lytic VP and VT averaged 7.3 × 10^9^ ± 4.2 × 10^9^ viruses l^-1^ d^-1^ and 0.6 ± 0.3 d^-1^, respectively (**Figures 11A,B**), and were within ranges previously reported for other marine ecosystems (Wilhelm et al., 2002; Poorvin et al., 2004; Winget et al., 2005; Weinbauer et al., 2009; Payet and Suttle, 2013). In general, VP and VT followed similar spatial trends along transects (**Figures 11A,B**), with highest and lowest values in the FRG and BR, respectively. Biotic (i.e., PA and BA) and abiotic (i.e., SRP, DIN and coordinates) variables explained 28 and 19% of spatial variability in VP, respectively, with LNA cells and SRP as the main predictors (**Table 2**). This implies phage infection of smaller bacteria may have been important, and is consistent with recent evidence showing phages are associated with highly abundant and small bacteria in the SAR11 and SAR116 clades in the oceans (Kang et al., 2013; Zhao et al., 2013).

Consistent with VP, VMB, BSSr and OCr displayed similar spatial trends across reef habitats (**Figures 10C–E**). On average, VMB removed 2.5 × 10^8^ ± 1.4 × 10^8^ bacteria l^-1^ d^-1^ (range: 5.1 × 10^7^–5.2 × 10^8^ bacteria l^-1^ d^-1^), which accounted for an estimated BSSr of 72 ± 31% d^-1^ (range: 24–133% d^-1^) and OCr of 4.9 ± 2.7 μg C l^-1^ d^-1^ (range: 1.0–10.4 μg C l^-1^ d^-1^) along cross-reef transects.

Assuming mean ambient DOC concentrations of 68 μM as previously measured in this reef ecosystem (e.g., Nelson et al., 2011), viral lysis may contribute to ca. 2–15% of the pool of DOC in these reef waters. This implies that viral lytic activities may be important in fueling labile organic carbon and associated nutrient supply to other non-infected microbes in these oligotrophic reef waters. This is especially true for the BR, which is known to be a particularly carbon depleted habitat (Nelson et al., 2011). Therefore, slow release of organic matter upon viral lysis maybe providing essential carbon and nutrient supply for microorganisms in the BR and sustain the low levels of heterotrophic and autotrophic microbes observed in this study.

For the time-series in the OB, VP, and VT estimates mirrored temporal patterns in VA and averaged 4.1 × 10^10^ ± 3.1 × 10^10^ viruses l^-1^ d^-1^ and 2.8 ± 2.4 d^-1^, respectively (**Figures 11B,C**). VP and VT displayed small temporal oscillations, with no significant differences detected between sampling dates (KW, *p* > 0.05). Temporal changes in biotic (i.e., PA and BA) and abiotic (i.e., SRP, DIN and time) variables explained 82 and 15% of variability VP, respectively, with APP, *Syn,* and SRP as the main predictors (**Table 2**). These results indicate that viral lytic production was strongly associated with changes in primary producers in the OB.

Consistent with VA and VP, higher estimates for VMB, BSSr, and OCr were detected in the OB compared to transects (**Figures 11B,C**). On average, VMB was responsible for the removal of 1.4 × 10^9^ ± 1.0 × 10^9^ bacteria l^-1^ d^-1^. This corresponds to an estimated BSSr of 152 ± 108% d^-1^ (range: 42–367% d^-1^) and OCr of 27.8 ± 19.6 μg C l^-1^ d^-1^ (range: 6.9–61.2 μg C l^-1^ d^-1^) in the OB. VMB, BSSr, and OCr followed similar temporal trends throughout the time-series, with relatively low date-to-date changes (**Figures 11B,C**). Similar to patterns in BA, PA and VA, these results suggest that viral lytic activity was relatively stable over time in the OB.

Again, assuming a mean DOC concentration of 68 μM (e.g., Nelson et al., 2011), these cellular lysis products were responsible for between 10 and 90% of ambient DOC levels in the OB. Thus, viral infection of heterotrophic bacteria may be an important source of DOC and associated nutrients for other non-infected microbes in the OB.

### METHODOLOGICAL CONSIDERATIONS

These results should be interpreted in the context of several limitations. We used FC to identify viral subsets, according to an established protocol that has been used in other studies (Brussaard et al., 2010). Although significant trends among viral subsets and biotic communities were detected, their identities still remain unclear. Recently, Martínez-Martínez et al. (2014) genetically characterized three viral subsets with relatively similar FC signatures to those reported in this study. While increased proportions of viruses infecting eukaryotic phytoplankton were detected from the V1 and V3 subset (Martínez-Martínez et al., 2014), these subsets still contained significant proportions of phages, highlighting the poor resolution of FC in distinguishing particular viruses associated with certain host cells. Thus, it is possible that this low resolution may have masked potential correlations among viruses and their hosts, explaining relatively weak relationships among viruses, biotic and abiotic variables. However, FC has become a standard methodology for studying of virus–host interactions, but can be extended and complemented by other microscopic techniques and molecular approaches.

During the viral-reduction approach, filtration steps required for reducing virus–host contact rates may have altered nutrient availability and microbial processes, potentially influencing results. In addition, estimates of BS and cellular carbon quota used to infer viral-induced mortality and carbon cycling are likely to fluctuate across gradients of microbial productivity. Despite these caveats, the viral-reduction approach has been successfully applied in various aquatic environments and has been shown to be a robust and straightforward approach for estimating viral lytic activity (Weinbauer et al., 2010b and references therein). The repeatable temporal patterns measured during our 3 week time-series study suggest that this viral-reduction approach can be applied to investigate lytic viral activity in response to variability in host abundance and environmental conditions.

Further work is needed to improve detection of viruses infecting microbial hosts in natural assemblages. In particular, the development of high-throughput methods to routinely detect virally infected microbial hosts from environmental samples, in conjunction with developments of specific molecular probes to target potential host–virus systems will provide novel insights on viral dynamics and their impacts in the oceans. Recently, the application of a new culture-dependent and independent approaches has allowed direct detection of viruses infecting microbial host isolates and offered new exciting perspectives for enabling simultaneous detection of host–virus interactions (Deng et al., 2012; Allers et al., 2013). However, the applicability to study a broader range of natural samples has still to be shown.

## SUMMARY

This study is the first to report the abundance, distribution and ecological impact of viruses in the coral reef waters of Moorea Island. Our data revealed distinct short-term spatiotemporal changes in VA and activity and demonstrated that these changes were linked to microbial host abundances and environmental variables. This work also confirmed general findings from other studies which have suggested that small shifts in host abundance and activity may be important in driving VA and lytic activity in marine systems (Fuhrman, 1999; Weinbauer, 2004; Seymour et al., 2005; Suttle, 2007; Brussaard et al., 2008; Clasen et al., 2008; Payet and Suttle, 2008; Rowe et al., 2008; Evans et al., 2009; Winget et al., 2011; Winter et al., 2012; Payet and Suttle, 2013).

Analysis of short-term temporal patterns in VA and lytic production in OB indicated persistent VA and infection. These findings confirm recent time-series studies that have also observed steady-state temporal dynamics of lytic viral activity (Winget and Wommack, 2009; Winget et al., 2011).

Highest VA and lytic activity as well as highest microbial host abundances were reported in FGRs as well as in OB, likely due to microgradients in nutrient availability.

Viral lysis was estimated to kill a significant fraction of heterotrophic microbes (%BSSr: 24–367%) daily. These mortality estimates are substantially higher than those estimated by Bouvy et al. (2012) in another reef ecosystem in French Polynesia, but were in agreement with other studies in other marine ecosystems (Wilhelm et al., 2002; Winget et al., 2005, 2011; Evans et al., 2009; Winget and Wommack, 2009; Evans and Brussaard, 2012; Payet and Suttle, 2013). Given that Bouvy et al. (2012) used frequency of visibly infected cells to infer mortality estimates through transmission electronic microscopy, it may be that this approach underestimated lytic viral impacts, as it heavily relies on specific conversion factors and potentially lacks of resolution due to sample preparations (e.g., see Weinbauer et al., 2002).

Notably, our data demonstrate that viral lysis substantially contributes to the overall pool of DOC (OCr: 1.0–62 μg C l^-1^ d^-1^) available to other microbes in these oligotrophic coral reef waters. Our estimates of OCr due to viral lysis were ca. 1- to 90-fold higher than previous reports in oligotrophic polar waters (Evans and Brussaard, 2012; Payet and Suttle, 2013), but within the range of other studies in marine environments (Wilhelm et al., 2002; Winget et al., 2005, 2011).

In conclusion, this study demonstrates that viruses have a key role in both top down and bottom up control of microbial communities in coral reef seawater.

## Conflict of Interest Statement

The authors declare that the research was conducted in the absence of any commercial or financial relationships that could be construed as a potential conflict of interest.
